# Left ventricle mass by cardiac magnetic resonance and echocardiography: the multi-ethnic study of atherosclerosis

**DOI:** 10.1186/1532-429X-14-S1-P296

**Published:** 2012-02-01

**Authors:** Anderson C Armstrong, Ola Gjesdal, Andre Almeida, Colin O Wu, Lyndia Brumback, Joao A Lima

**Affiliations:** 1 Cardiovascular Imaging, Johns Hopkins Hospital, Baltimore, MD, USA; 2 Cardiology, Universidade Federal do Vale do São Francisco, Petrolina, Brazil; 3 Biostatistics, University of Washington, Seattle, WA, USA; 4 OD/NHLBI/NIH, Bethesda, MD, USA

## Background

Cardiac magnetic resonance (CMR) is the gold standard imaging method to assess left ventricular mass (LVM), but M-mode echocardiography is more common during clinical practice. Proposed methods to normalize LVM to anthropometric measures provide different cut-off values for LV hypertrophy (LVH). We compare LVM assessed by echocardiography and CMR and evaluate the level of agreement for classification of LVH.

## Methods

A randomized subsample of African-American and Caucasian participants free of clinical cardiovascular disease from the Multi-Ethnic Study of Atherosclerosis (MESA) had echocardiography and CMR performed on the same day at the Johns Hopkins Hospital (JHH), Baltimore, MD. LVM was assessed by echocardiography as recommended by the American Society of Echocardiography. By CMR, short-axis images covering the entire LV were acquired; the difference between epicardial and endocardial areas for all slices was multiplied by the slice thickness (6 mm) and section gap (4mm), and then multiplied by the specific gravity of myocardium (1.04 g/ml), with exclusion of papillary muscle mass. LVH was defined according to normalization by body surface area (BSA), height^1.7^, height^2.7^, or by the expected LVM (95th percentile of subjects free of co-morbidities). LVM assessments were compared using Pearson’s correlation, intraclass correlation coefficient (ICC), and Bland-Altman plots. The percent agreement and the Cohen’s Kappa coefficient were calculated to evaluate the agreement of LVH classification.

## Results

**Figure 1:**
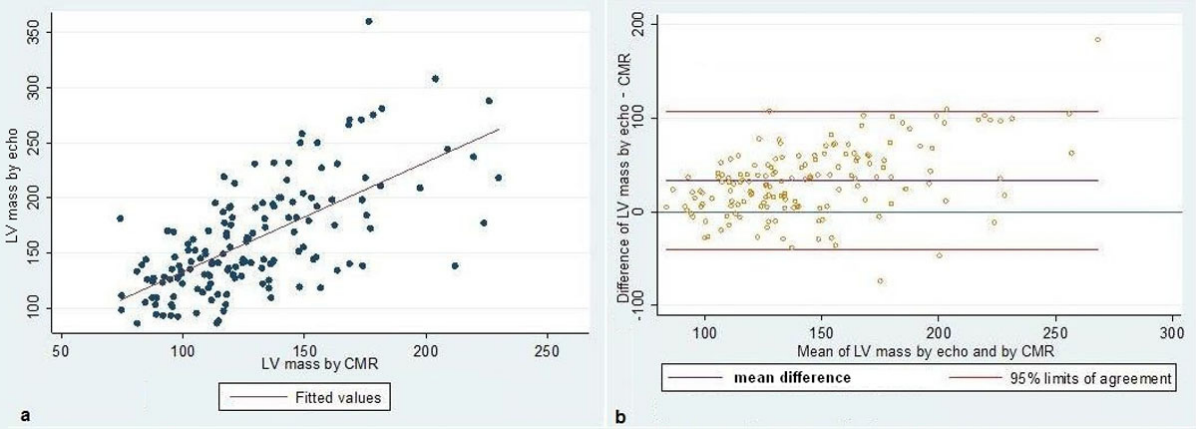
LVM assessed by echocardiography and CMR: scatter plot for linear correlation (a) and Bland-Altman plots for agreement (b).

A total of 155 MESA participants (66.3±8.9 years; 41% males; 53.1% Caucasian) were included. A moderately strong correlation was found between LVM assessed by echocardiography and CMR (r=0.66, p<0.001, Fig.1a), the ICC was 0.48 (95% CI 0.29, 0.68). The Bland-Altman plot demonstrated overestimation of LVM by echocardiography relative to CMR (33.1g, 95% CI -40.6, 106.7g, Figure 1b). CMR assessment demonstrated 5.8% LVH when normalized by BSA; 6.4% by height^1.7^; 5.8% by height^2.7^; and 3.2% by expected LVM. Assessed by echocardiography, 28.4% had LVH normalized by BSA; 38.7% by height^1.7^; 23.2% by height^2.7^; and 17.4% by expected LVM. The percent of agreed classification ranged from 93 to 97% using CMR-derived parameters; 79 to 94% using echocardiographic assessment; and 59 to 81% between CMR and echocardiographic assessments. The Kappa coefficient for comparing CMR and echocardiography was statistically significant only when indexed by BSA (table 1).

**Table 1:** Proportion of agreed classification (below diagonal) and Kappa coefficient of agreement (above diagonal) for the classification of LV hypertrophy according to image methodology and normalization procedure.

Normalization Methodology		Cardiac Magnetic Resonance				Echocardiography			
	
		BSA	Expected	height^1.7	height^2.7	BSA	height^1.7	height^2.7	Expected
CMR	BSA	*	0.70†	0.38†	0.41†	0.10§	0.017	0.09	0.03
	Expected	97%	*	0.37†	0.55†	-0.02	0.002	0.04	0.008
	height^1.7	93%	94%	*	0.61†	-0.35	-0.03	-0.02	-0.04
	height^2.7	94%	96%	96%	*	-0.02	0.02	0.04	0.02

Echo	BSA	72%	70%	68%	68%	*	0.57†	0.76†	0.70†
	height^1.7	61%	61%	59%	61%	81%	*	0.62†	0.50†
	height^2.7	76%	76%	73%	75%	91%	83%	*	0.82†
	Expected	79%	81%	77%	79%	89%	79%	94%	*

CMR - Cardiac Magnetic Resonance; Echo - Echocardiography †p<0.0001 ; § p < 0.05

## Conclusions

LV mass assessed by echocardiography and CMR demonstrated good correlation. The prevalence of LVH was lower when assessed by CMR. Higher agreement was present when the techniques were compared separately and lower for comparisons between echocardiography and CMR. Due to importance of myocardial hypertrophy for clinical and research purposes, standardization of the assessment by CMR, by echocardiography, and across modalities is encouraged.

## Funding

National Heart,Lung, and Blood Institute.

